# Risperidone-Induced Urinary Retention: A Case Report Highlighting Urological Complications in Long-Term Use of Antipsychotic Medication

**DOI:** 10.7759/cureus.85102

**Published:** 2025-05-30

**Authors:** Khalifa Bin Zaal, Ammar Agha, Amr Elmekresh, Shaima M Abuhejleh, Elham Mahjoor Azad, Yaser Saeedi, Fariborz Bagheri

**Affiliations:** 1 Urology, Dubai Hospital, Dubai, ARE; 2 Family Medicine, Medcare Hospital, Dubai, ARE; 3 Urology, Dubai Health, Dubai, ARE

**Keywords:** atypical antipsychotics, bladder wall thickening, chronic cystitis, risperidone, urinary retention

## Abstract

Urinary retention is a urological emergency that can lead to serious complications if left untreated. Although rare, risperidone has been associated with urinary retention through anticholinergic effects and modulation of dopaminergic and serotonergic pathways, which can inhibit detrusor muscle contractility and increase urethral sphincter activity. This report presents the case of a 54-year-old male patient who experienced multiple episodes of urinary retention that resulted in gross hematuria, hydronephrosis, and hospital admissions; this was considered to be caused by risperidone, which he was taking for bipolar disorder. This case highlights the importance of considering medication side effects in patients with recurrent urological issues, particularly those with multiple comorbidities. By contributing to the limited literature on risperidone-induced urinary retention, this report aims to enhance clinical awareness and guide decision-making in similar presentations.

## Introduction

Urinary retention is a urological emergency and, if left untreated, it could lead to serious renal injury or urosepsis [1]. This presentation can be due to lower urinary tract obstruction, inflammation, neurogenic, or iatrogenic causes [2]. We report the case of a patient with a history of bipolar disorder, who was prescribed risperidone, which is a benzoxazole derivative used to treat this condition [3], for a duration of six months before the presentation. We hypothesize that the long-term use of risperidone could be the cause of urinary retention in this patient, a theory that has not been extensively explored in the literature. This case report aims to discuss this potential association in greater depth, contributing to the understanding of antipsychotic medication-related urological complications.

## Case presentation

A 54-year-old male patient presented with recurrent episodes of urinary retention. His medical history included type 2 diabetes mellitus (T2DM) and a diagnosis of bipolar disorder with aggressive behavior. He had been treated with risperidone monotherapy (2-3 mg daily) intermittently for the previous 2.5 years and had been taking it continuously for the past six months. No other psychiatric medications were reported. Unfortunately, there was no reliable history regarding the total duration of his psychiatric illness or the number of manic or depressive episodes. The patient had frequent hospital admissions over the past year, primarily due to lower urinary tract symptoms (LUTS) and urinary retention. 

**Figure 1:**
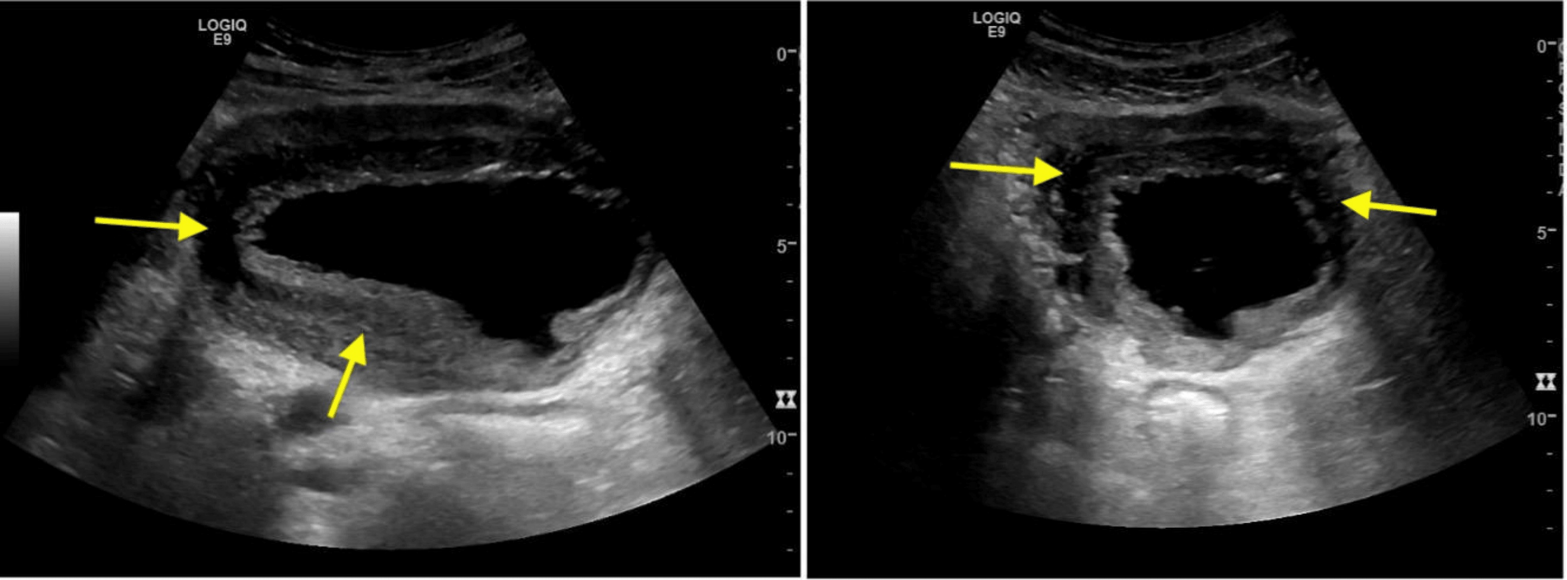
Suboptimally distended bladder, showing extensive irregular circumferential wall thickening

At this presentation, he reported gross haematuria and suprapubic pain. He was hemodynamically stable with normal inflammatory markers and a creatinine level of 1.0 mg/dL (Table 1). Ultrasound revealed bilateral hydronephrosis, diffuse bladder wall thickening, and an enlarged prostate measuring approximately 45 cc (Figure 1). Catheterization relieved his retention.

**Table 1:** Laboratory results on presentation These unremarkable findings reduce the likelihood of infection or renal dysfunction as immediate causes of retention.

Parameters	Patient Values	Units	Reference Ranges
White blood cell count	8.8	×10^3^/µL	3.6 – 11
Red blood cell count	4.28	×10^6^/µL	4.5 – 5.5
Hemoglobin	12.2	g/dL	13 – 17
Platelet count	193	×10^3^/µL	150 – 410
C-reactive protein	0.6	mg/L	< 5.0
Procalcitonin	0.03	Ng/mL	< 0.05
Creatinine	1.0	mg/dL	0.7 – 1.2
Glomerular filtration rate	90.6	mL/minute/1.73m^2^	> 60

Four days after presentation, cystourethroscopy revealed an erythematous bladder wall with vesicle-like lesions. A transurethral resection of a bladder tumor (TURBT) was performed. Histopathology showed severe polypoid/papillary hemorrhagic chronic cystitis with hyperplastic, focally inflamed, and eroded urothelium. No malignancy was identified.

One month later, the patient re-presented with acute urinary retention, elevated creatinine, and increased inflammatory markers. A CT abdomen revealed a distended bladder with cystitis and upper tract changes, including bilateral hydroureter and hydronephrosis. No stones were found. Benign prostatic hyperplasia (BPH) was noted radiologically, although no obstructive pattern was evident on urodynamic or physical examination.

He underwent bilateral ureteric stent placement and a repeat TURBT. Symptoms persisted, leading to the insertion of a suprapubic catheter. This resulted in clinical stabilization, with preserved renal function and no further episodes of acute retention reported at the six-month follow-up.

## Discussion

This case highlights a possible rare adverse effect of risperidone contributing to chronic urinary retention. While urinary retention is typically associated with obstructive, infectious, or neurologic etiologies [4,5], medication-induced retention, especially from antipsychotic medication, should be considered.

Risperidone, an atypical antipsychotic medication, is widely used to treat schizophrenia and bipolar disorder [6]. Although not commonly linked to genitourinary side effects, case reports suggest that risperidone can cause urinary retention in approximately 0.3-0.5% of users [7]. In our literature review, fewer than 10 individual case reports describe risperidone-induced urinary dysfunction [8-14]. Most involved transient retention that resolved after medication withdrawal.

In this patient, BPH was radiologically present but unlikely to be the primary cause, as repeated imaging and clinical assessments failed to demonstrate a clear outlet obstruction. Diabetic cystopathy, typically causing overflow incontinence rather than acute retention, was also considered but unsupported by symptoms or urodynamic data. Neurological causes were excluded based on a normal exam and no history of stroke or spinal pathology.

Risperidone may impair bladder function through multiple mechanisms. It antagonizes D2 receptors and 5-HT2A/C receptors, which regulate bladder detrusor activity. This reduces contractility and increases urethral sphincter tone, impairing voiding [15]. Additionally, serotonin antagonism may dysregulate dopaminergic control of micturition-5-HT2A inhibition increases dopamine D1 activity, suppressing overactive bladder, while D2 activation may paradoxically worsen detrusor overactivity [16,17].

The observed bladder wall thickening is a radiological sign suggestive of chronic inflammation or muscular hypertrophy due to repeated urinary retention. While not a diagnosis, it warrants further evaluation to identify contributing factors. In this case, histology confirmed chronic cystitis without neoplasia.

Importantly, risperidone was continued throughout the patient's management. Despite this, the patient showed clinical improvement after suprapubic catheter insertion, with no further episodes of urinary retention and resolution of hydronephrosis noted on follow-up imaging. While this limits the ability to definitively attribute urinary retention to risperidone, the persistent symptoms despite anatomical interventions and the absence of other clear etiologies suggest that risperidone may have played a contributory role.

## Conclusions

In this report, we highlighted the interplay between psychiatric medication and urological side effects. Risperidone is an effective medication in treating bipolar disorder, although it is rare, but it has a potential role in contributing to urinary retention. This patient’s presentation shows the importance of frequent evaluation of patients on antipsychotic medications, such as risperidone, by urologists for any voiding dysfunction to prevent complications like hydronephrosis or a thickened bladder wall. This report adds to the limited literature on risperidone-induced urinary retention and emphasizes the need for a multidisciplinary approach in managing such cases. Further research is essential for a better understanding of the mechanisms underlying antipsychotic medication-related urological complications and to guide clinical decision-making in patients with similar presentations.
